# Still puzzling questions in immunology (infection and immunity)

**DOI:** 10.1186/s12865-015-0084-1

**Published:** 2015-03-26

**Authors:** Olivier Garraud, James Li

**Affiliations:** EA3064—Faculté de Médecine, Université de Lyon, 42023 Saint-Etienne, France; Institut National de la Transfusion Sanguine, 75015 Paris, France; Department of Paediatrics and Adolescent Medicine, LKS Faculty of Medicine, The University of Hong Kong, Pok Fu Lam, Hong Kong

During the last decade, microbial science was one of the hottest topics in scientific research with the number of publications in this field nearly doubling every 5 years. The results of these studies help to bring us the development of hundreds of vaccines and drugs for human and veterinary use. The results of our research not only protect humans they also help to provide stable food supply to our community. Meanwhile, we have been continuously—and we still are—under threat from new emerging infections such as avian influenza, new seasonal flu, SARS and recently the Ebola virus; Dengue virus infection is forecasted to develop worldwide, posing novel threats. In addition, other pathogenic microbes such as HIV, *Mycobacterium tuberculosis*, and antibiotic resistant bacteria that are widespread in parts of our population further threaten our safety. Currently, scientists and clinicians are working closely together to develop new treatments to fight against these infections; however, questions are continuously raised to understand how the immune system that happens to be defeated by a number of dangerous pathogens can be manipulated to combat such infections.

Our immune system is indeed designed to protect us from invaders.

- Innate immunity is the first line of defense against infection. There have been amazing discoveries in the field of innate immunity, with lots of newly discovered cells or cell subsets that have a crucial function but innate immunity alone is not enough for us to get rid of infections. More precisely, we still do not fully understand how these pathogens activate our immune system and how our immune system fights against pathogens. After the early innate immune responses, the body develops the adaptive immunity to protect ourselves from future infections.
- Our knowledge on adaptive immunity is also limited, especially on the generation of antibodies. Furthermore, the genetics of antigen presentation and genetic susceptibility to infections appear seminal, but are not fully comprehended.
- The study of intracellular parasites and mycobacteria helped open the door to modern immunology back in the 90’s, leading to the discovery of functionally distinct helper T cell subpopulations Th_0_, Th_1_, Th_2_. Furthermore, we have recently discovered more immune cell subsets including Th_FH_, Th_9_, Th_17_, T_Regs_, along with the non-T lymphocyte effectors and regulators. However, the fight against both types of infectious pathogens (intracellular parasites and mycobacteria) has poorly benefited from the tremendous progress made in immunology. We are no closer to containing the spread of these pathogens in our community, as we are still far from limitation and even more far from eradication. Our modern immunology knowledge has helped us to graft kidneys, hearts and lungs, livers, intestines, and even hands and faces, but we still fail in combating the major five killers regarding infectious diseases i.e. lower respiratory infections, diarrheal diseases, HIV/AIDS, tuberculosis and malaria. Although these diseases are usually found in developing countries, we also need to pay more attention, just like the Ebola pandemic, as it can easily spread and become a global problem if not properly dealt with.
- Vaccines are our ultimate weapons against microbial infection. However, in spite of all our efforts in vaccine development and more than 120 years after the first human vaccine against the rabies virus, there are still some very disappointing failures in the development of efficient vaccines against some major infectious killers and debilitating pathogens. In 1967, one of the leading journals, Nature (London), claimed the discovery of a vaccine against malaria. The fact is, after 50 years of effort, a malaria vaccine has still not been approved for human use. Another famous example is the development of the HIV vaccine. Since 1983, scientists and clinicians have investigated the treatment of AIDS. After years of effort, different anti-viral drugs and regimes have been successfully developed in order to control AIDS progression. However, although a huge amount of funding has been spent, the progress of the development of a successful HIV vaccine is still lagging far behind public expectations. Apart from vaccine development for newly identified microbes, there are also difficulties faced in developing improved vaccines against some pathogens. Tuberculosis is a disease caused by *M. tuberculosis*. A hundred years ago, BCG was developed as the vaccine for tuberculosis. Due to its limited efficacy on the protection against tuberculosis, there is high expectancy that a suitable vaccine would replace the canonical BCG vaccine. Despite its extremely debated shortfalls, the BCG vaccine is still widely used in many healthcare systems to combat the spread of tuberculosis and leprosy, which is another complication that has suffered from a lack of both efficient therapeutic and preventive means against mycobacterium infection.
- Due to a number of unsuccessful vaccine design trials targeting major epitopes of infectious agents, scientists are starting to develop other strategies to defend the host. The current trend on vaccine design and adjuvant development is to either activate the host immune system to fight against pathogens or to exhaust specific as well as non-specific immune responses to dampen diseases, such as autoimmune diseases and cancer. In addition, there are several vaccines undergoing clinical trials that are targeting both the pathogens themselves and also boosting the immune response specific to an infection. Autoimmune disorders and cancer would not be expected to be questioned in this “series” dedicated to “Infection and Immunity”; however, novel findings in immunology re-emphasizes on the role of infectious triggers, acute and then chronic inflammation, and chronic state pathogenesis and immune impairment: a look back to latent infection and how it affects early immune responses may give new directions for future care and cure of patients.
- Another puzzling issue in the world currently is the incredible increase of patients with mild or severe allergic-type symptoms. Allergy affects billions of people worldwide and can be life threatening. Allergy can be caused by numerous environmental factors such as air pollution and various chemical; participation of infectious triggers—present or absent—is still debated in the constitution of atopic states (the hygiene theory): this demonstrates that our immune system may have to be trained (in particular to face microbes, that are pathogen or not) in order to gain optimal performance; this also calls for a revisit of the pathogenicity of microbes and—at large—of the role of the microbial ecology of the human being(s), to maintain a certain equilibrium and avoid emergent infectious outbreaks.
- Lastly, because this is a fast expanding universe—though linked to the above mentioned item relative to microbial ecology—, more research should also be focused on the precise role of novel immune organs such as the microbiota, which is envisioned to dialog with the environment; this is expected to allow a revisit of inflammatory bowel diseases and treatment regimens, that may now start with viral (CMV) cure prior to immunomodulating agents.

This series of forum articles in “BMC Immunology” is thus aimed at addressing debatable questions in the field of infection and immunity, with the hope of promoting research to further our understanding of the complicated immune system and ultimately, to provide benefits to humans while not altering their ecology.

